# Draft Genome Sequence of *Fusarium fujikuroi* B14, the Causal Agent of the Bakanae Disease of Rice

**DOI:** 10.1128/genomeA.00035-13

**Published:** 2013-02-28

**Authors:** Haeyoung Jeong, Seunghoon Lee, Gyung Ja Choi, Theresa Lee, Sung-Hwan Yun

**Affiliations:** a Industrial Biotechnology & Bioenergy Research Center, KRIBB, Daejeon, South Korea; b Department of Medical Biotechnology, Soonchunhyang University, Asan, South Korea; c Chemical Biotechnology Research Center, KRICT, Daejon, South Korea; d Microbial Safety Team, National Academy of Agricultural Science, RDA, Suwon, South Korea

## Abstract

Here, we present the genome sequence of a Korean strain (B14) of *Fusarium fujikuroi*, a fungal rice pathogen. The final assembly consists of 455 contigs with 43,810,516 bp and 14,017 predicted genes. Comparison with the *F. verticillioides* 7600 genome revealed a reference coverage of 83% (66.3% of reads mapped).

## GENOME ANNOUNCEMENT

The genus *Fusarium*, a large group of filamentous ascomycetous fungi, includes a broad range of plant pathogens in many agricultural crops worldwide (1). Some species produce mycotoxins harmful to both humans and plants (2). Thus far, genomes of three species (*F. graminearum, F. oxysporum*, and *F. verticillioides*) have been sequenced (3–5). *F. fujikuroi* (teleomorph, *Gibberella fujikuroi*; synonym, *G. fujikuroi* mating population C) is a biologically and phylogenetically distinct species within the *G. fujikuroi* species complex and causes bakanae disease of rice (1, 6). This fungus also produces several toxic secondary metabolites in infected plants (1). Here, we present the genome sequence of *F. fujikuroi* (strain B14) isolated from rice in South Korea and confirm its pathogenicity in rice. This is the first available *F. fujikuroi* whole-genome sequence and the second, after the *F. verticillioides* 7600 strain, among the *G. fujikuroi* complex.

Genome sequencing of *F. fujikuroi* B14 was carried out using an Illumina HiSeq 2000-based whole-genome shotgun strategy. A total of 35,306,706 paired-end reads of ~3.57 Gb (101 nucleotide [nt] cycle, 486-bp average paired distance) were preprocessed and *de novo* assembled using CLC Genomics workbench 5.5. Initially, the assembly was 43,794,120 bp in length with 338 scaffolds (N_50_ 678,621 bp, 48.3% G+C, 1,079 contigs). After automatic gap closing using GapFiller version 1.9 (http://www.baseclear.com) with the same reads, the final assembly consisted of 455 contigs in 333 scaffolds with a length of 43,810,516 bp exclusive of N’s in remaining gaps. After masking repetitive sequences using a search against Repbase (http://www.girinst.org/repbase), 14,017 protein-coding genes were predicted using Augustus 2.5.5 (http://augustus.gobics.de) with *F. graminearum* parameters. Based on a BLASTP search against the UniRef90 database, significant matches (E value <10^–5^) were identified for 13,734 genes; 9,143 hits were derived from *F. oxysporum*. We also identified 576 tRNA genes using tRNAscan-SE (7). For comparative genomic analysis, the preprocessed Illumina reads were mapped to chromosomal reference sequences for the three known *Fusarium* species (http://www.broadinstitute.org/annotation/genome/fusarium_group/MultiHome.html). *F. verticillioides* 7600 was most similar to B14 in terms of reference coverage (83%; 66.3% of reads were mapped). The percent coverage values of *F. oxysporum* 4287 and *F. graminearum* PH-1 were 57% and 29%, respectively. BLASTP analysis showed that 46.2% and 42.1% of the B14 genes matched those of *F. oyxsporum* (total, 17,701 genes) and *F. verticillioides* (14,188 genes), respectively. In the B14 genome, all conserved gene clusters for secondary metabolites previously characterized in *F. fujikuroi* were identified, including those for gibberellin, fumonisin, bikaverin, melanin, fusarin, fusaric acid, and carotenoids. Additionally, *F. fujikuroi* B14, confirmed as the *MAT1-2* mating type strain, carries 20 polyketide synthase (PKS) genes, including two nonreducing *PKS* and four *PKS-NRPS* (non-ribosomal peptide synthetase) hybrid genes.

In conclusion, the *F. fujkuroi* B14 genome will contribute to a greater understanding of the biology and evolution of the *G. fujikuroi* species complex, as well as the genus *Fusarium*.

### Nucleotide sequence accession number.

The sequence determined in this study was deposited in the GenBank database under accession number ANFV00000000.
